# Draft Genome Sequence of Norvancomycin-Producing Strain *Amycolatopsis orientalis* CPCC200066

**DOI:** 10.1128/genomeA.00296-15

**Published:** 2015-05-14

**Authors:** Xuan Lei, Fang Yuan, Yuanyuan Shi, Xingxing Li, Lifei Wang, Bin Hong

**Affiliations:** Key Laboratory of Biotechnology of Antibiotics of Ministry of Health, Institute of Medicinal Biotechnology, Chinese Academy of Medical Sciences and Peking Union Medical College, Beijing, China

## Abstract

*Amycolatopsis orientalis* CPCC200066 is an actinomycete that can produce the glycopeptide antibiotic norvancomycin, which has significant inhibitory activity against Gram-positive cocci and bacilli. Here, we report the draft genome sequence of *A. orientalis* CPCC200066 and identified the genes involved in norvancomycin biosynthesis.

## GENOME ANNOUNCEMENT

*Amycolatopsis orientalis* CPCC200066 (B-37) was initially isolated from a soil sample in Guizhou Province, China, in 1959 (1) and was obtained by the China Pharmaceutical Culture Collection (CPCC). It produces the glycopeptide antibiotic norvancomycin, whose chemical structure is almost the same as that of vancomycin, except for an absent methyl group at the N terminus. Norvancomycin shows pharmacological properties and antibacterial activities similar to those of vancomycin, which exhibits significant inhibitory activity against Gram-positive cocci and bacilli*,* especially to methicillin-resistant *Staphylococcus aureus* (MRSA) and methicillin-resistant *Staphylococcus epidermidis* (MRSE). Due to its potential clinical application, norvancomycin was commercially developed by the North China Pharmaceutical Company and has been widely used in China to treat endocarditis, osteomyelitis, and other severe infections caused by *S. aureus* (including methicillin-resistant strains) (2) for >3 decades. Genome sequencing of *A. orientalis* CPCC200066 may provide information for discovering the biosynthetic gene cluster for norvancomycin. Here, we present a draft genome sequence of *A. orientalis* CPCC200066.

The strain was grown at 28°C in tryptic soy broth (TSB) liquid medium (2% tryptone, 0.5% NaCl, 0.25% glucose, 0.25% K_2_HPO_4_ [pH 7.2]), and genomic DNA was extracted using the DNA extraction kit (TANBead, China). Sequencing was performed using an Illumina HiSeq 2000 platform at the Beijing Genomics Institute (BGI) (Shenzhen, China), resulting in 14,002,362 reads with 39-fold average coverage. Short reads were assembled by SOAP*denovo* 2.04 (3), and Glimmer 3.02 was used to predict protein-coding sequences (CDSs) (4). The draft genome sequence was annotated based on the KEGG, COG, SwissProt, NR, GO, and PHI databases.

The draft genome sequence of *A. orientalis* CPCC200066 is 9,438,289 bp, with a G+C content of 68.85% distributed over 59 scaffolds containing 88 contigs. The *N*_50_ length of the scaffolds was 402,393 bp, and that of the contigs was 246,913 bp. A total of 8,233 CDSs were predicted, with an 86.36% coding density and a 990-bp average length. For these CDSs, >300 proteins were identified as being involved in secondary metabolism, and this will help us to explore novel secondary metabolites in *A. orientalis* CPCC200066. The gene cluster responsible for norvancomycin biosynthesis was assigned on scaffold 20 and consists of 30 genes, which shows a high similarity with reported gene clusters of the glycopeptide antibiotics chloroeremomycin (5, 6) and vancomycin (7, 8). Consistent with the chemical structure difference of lacking one vancosamine compared to chloroeremomycin, the norvancomycin biosynthetic gene cluster contained only two glycosyltransferase genes compared with three in the chloroeremomycin cluster. Interestingly, one *N*-methylase gene was identified in the norvancomycin biosynthetic gene cluster as in the vancomycin biosynthetic clusters, while the main vancomycin analogue of CPCC200066 is absent of an *N*-methyl group. The function of the *N*-methylase gene in the norvancomycin biosynthetic gene cluster and the biosynthetic mechanism of norvancomycin in CPCC200066 warrant further investigation. The genome sequence of CPCC200066 will aid in further studies for designing strategies for the construction of strains with enhanced norvancomycin production and the discovery of new natural products by uncovering cryptic metabolic pathways.

### Nucleotide sequence accession numbers. 

The whole-genome shotgun project has been deposited in DDBJ/EMBL/GenBank under the accession no. JXRD00000000. The version described in this paper is the first version, JXRD01000000.
